# Losing a child to adolescent cancer: A register‐based cohort study of psychotropic medication use in bereaved parents

**DOI:** 10.1002/cam4.5347

**Published:** 2022-10-11

**Authors:** Emma Hovén, Lisa Ljungman, Josefin Sveen, Charlotte Skoglund, Gustaf Ljungman, Rickard Ljung, Anna Wikman

**Affiliations:** ^1^ Department of Women's and Children's Health Uppsala University Uppsala Sweden; ^2^ Childhood Cancer Research Unit, Department of Women's and Children's Health Karolinska Institutet Stockholm Sweden; ^3^ Center for Crisis Psychology University of Bergen Bergen Norway; ^4^ Department of Clinical Neuroscience Karolinska Institutet Stockholm Sweden; ^5^ Pediatric Oncology Uppsala University Hospital Uppsala Sweden; ^6^ Unit of Epidemiology Institute of Environmental Medicine, Karolinska Institutet Stockholm Sweden

**Keywords:** adolescence, bereavement, cancer, parents, psychotropic medication

## Abstract

**Purpose:**

To investigate the short‐ and long‐term risk of psychotropic medication use in parents who lose a child to cancer diagnosed in adolescence.

**Methods:**

This is a Swedish nationwide register‐based study including 184 bereaved mothers and 184 bereaved fathers of 184 children diagnosed with cancer in adolescence. Logistic regression analyses, adjusted for sociodemographic characteristics and history of mental health problems, were performed to estimate risk of a prescription of psychotropic medication (anxiolytics, hypnotics/sedatives, antidepressants) in cancer‐bereaved parents from 1 year before to 5 years after the child's death, with a general population sample of non‐bereaved parents (*n* = 3291) as referents.

**Results:**

At the year of the child's death, 28%–36% of mothers and 11%–20% of fathers had a prescription of anxiolytics, hypnotics/sedatives or antidepressants. The corresponding percentages for non‐bereaved mothers and fathers were 7%–12% and 4%–7%, respectively. Compared to non‐bereaved mothers, bereaved mothers showed higher odds of prescriptions from 1 year before up to four (anxiolytics) and 5 years (hypnotics/sedatives and antidepressants) after the child's death. Bereaved fathers showed higher odds than non‐bereaved fathers of prescriptions from 1 year before up to the year of (anxiolytics and hypnotics/sedatives) and 1 year after (antidepressants) the child's death. No differences in odds between bereaved and non‐bereaved fathers were found at 2 years after the child's death. Being unmarried, born outside Sweden, and having a history of mental health problems were associated with higher odds of prescribed medications.

**Conclusions:**

Indicative of mental health problems of clinical importance, cancer‐bereaved parents had a higher prevalence of use of psychotropic medication. A decrease in medication use was evident with time, but still at 5 years after the child's death mothers displayed a higher use while fathers showed no difference to non‐bereaved fathers after 2 years.

## 1 INTRODUCTION

Losing a child is one of the most terrifying and overwhelming experiences for a parent. Despite major treatment advances in recent decades, about one fifth of parents of children diagnosed with cancer will experience the death of their child.^1^, ^2^ Bereavement is associated with various mental health problems, including loneliness, suicidal ideation, insomnia, depression, and anxiety.^3^ It has been reported that grief after the loss of a child is more intense and persistent than after other types of bereavement, for example loss of a partner or parent.^4^ Bereaved parents have been found to be at risk for adverse psychological health,^5^, ^6^, ^7^ hospitalization for mental illness,^8^ morbidity,^9^ and mortality.^10^, ^11^

Psychotropic medications are used to treat a variety of mental health issues that cause significant impairment to healthy functioning. Psychotropic medications are only available on prescription by a medical doctor in Sweden, thus providing an objective measure of underlying mental health problems. A previous register‐based study identified an increased use of antidepressants and anxiolytics in parents who have lost a child (to external cause or disease) compared to parents in the general population, with stronger adverse impact of a child's death on mother's mental health.^12^ To date, much of the data on cancer‐bereaved parents have been derived from retrospective, cross‐sectional self‐report studies.^7^, ^13^, ^14^, ^15^, ^16^, ^17^ It has been confirmed that parents who lose a child to cancer report more adverse mental health symptoms, for example posttraumatic stress, anxiety and depression, relative to parents of childhood cancer survivors and normative population data.^17^, ^18^, ^19^, ^20^ Still, the need for rigorous, prospective research using objective measures to investigate parents' mental health following the death of a child to cancer has been put forth.^17^

To the best of our knowledge, only one study has used register data to report on psychotropic medication use in parents of children with cancer.^21^ Results showed an increased risk of a first prescription of anxiolytics and hypnotics/sedatives following a child's cancer and highlight that bereaved parents (pooled data for mothers and fathers) are at particular risk. Further investigation is needed to strengthen the empirical evidence on psychotropic medication use when losing a child to cancer. While studies support that cancer‐bereaved parents experience significant psychological sequelae, the pattern of use of psychotropic medications in mothers and fathers, respectively, has not been examined. Moreover, compared to parents who lose their children in more sudden, unexpected deaths due to external causes, cancer‐bereaved parents are often exposed to lingering emotional and physical suffering of their children,^22^ which makes it important to study also the time preceding the child's death.

This study is part of a larger research project on psychiatric consequences of adolescent cancer.^23^, ^24^ A diagnosis of cancer in adolescence occurs at a phase of physical, cognitive, psychological, and social development. In addition to existing adolescent developmental challenges, adolescents with cancer and their parents are faced with specific cancer‐related stressors, including restrictions in activity, isolation from healthy peers, increased dependency on parents, and changes in physical appearance.^25^, ^26^, ^27^ In view of that, adolescents with cancer have been described as a distinct subgroup of patients,^26^ for which patient and parent outcomes should be acknowledged specifically. Furthermore, grief among parents who had lost a child up to the age of 17 has been found to increase with the age of the child.^28^ To examine cancer‐bereavement outcomes of parents of adolescents diagnosed with cancer thus appears particularly important.

In this nation‐wide register‐based cohort study, we investigated whether the death of a child to cancer diagnosed in adolescence is associated with an increased risk of psychotropic medication (antidepressants, anxiolytics, or hypnotics/sedatives) use, taking into consideration sociodemographic characteristics and the time since the child's death. A specific aim was to identify if and when the level of use of psychotropic medication is comparable to a general population sample of non‐bereaved parents.

## 2 METHODS

### 2.1 Study sample

All adolescents (*n* = 2822) who were born in Sweden from 1980 to 2003, registered as residents in Sweden at age 12, and had received a first cancer diagnosis in adolescence (13–19 years) were identified via the Swedish Cancer Register,^23^ with an overall completeness of at least 96%.^29^ The selection of participants born 1980 until 1993 was made based on the completeness and availability of data in the national registries. Moreover, different definitions of adolescence has been used in psycho‐oncological research,^27^, ^30^, ^31^ and while the World Health Organization defines adolescence as the period from ages 10–19,^32^ our data were restricted to adolescents diagnosed between the ages of 13–19. To allow for analyses of psychotropic medication prescriptions 1 year prior to the child's death and with a follow‐up time of at least 2 years (end of follow‐up 31 December 2016), only parents who lost their child between 1 July 2006 and 31 December 2014 were included. Using the Swedish Cause of Death Register,^33^ 184 adolescents were identified to have died of cancer‐related causes during this time period.

A randomly selected comparison group (ratio 1:10), free of childhood cancer matched to the adolescents with cancer on age, sex, and geographical location (county of residence at the date of diagnosis), was identified from the Total Population Register.^34^ The Multi‐generation register,^35^ was used to identify biological parents of the adolescents with cancer and biological non‐bereaved parents of the cancer‐free comparisons.

### 2.2 Measures

#### 2.2.1 Outcomes

Information on prescribed psychotropic medication was obtained from the Swedish Prescribed Drug Register,^36^ established on 1 July 2005, which includes information on all medications prescribed in Sweden, classified according to the Anatomic Therapeutic Chemical Classification System (ATC).^37^ The following medications were analyzed: anxiolytics (N05B), hypnotics/sedatives (N05C), and antidepressants (N06A).

#### 2.2.2 Covariates

Sociodemographic variables (age, education, marital status, country of birth) were collected from the Longitudinal Integrated Database for Health Insurance and Labour Market Studies.^38^ History of severe mental health problems was obtained from the Swedish Patient Register,^39^ defined as any in‐ or out‐patient psychiatric diagnosis during the 5 years prior to date of the child's cancer diagnosis. The Swedish Patient Register contains information on all psychiatric in‐ and specialized out‐patient diagnoses in Sweden, with all specialized out‐patient care since 2001 and complete nationwide in‐patient coverage since 1987. Diagnoses are recorded according to the International Statistical Classification of Diseases and Related Health Problems (ICD).^40^ The following ICD codes (primary diagnosis) were included: F01‐F99, X60‐X84, Y10‐Y34 (ICD‐10), and 290–319, E950‐E959, E980‐E989 (ICD‐9).

### 2.3 Analyses

Logistic regression analyses were performed using generalized estimating equations (GEE) to estimate risk of psychotropic medication use in cancer‐bereaved parents, with non‐bereaved parents of cancer‐free comparisons as referents. An autoregressive correlation structure was chosen to account for measurements from the same individual being more correlated with each other the closer in time measurements are spaced. Odds ratios (OR) and 95% confidence intervals (CI) were recorded for crude (Model 1) and adjusted models (Models 2 and 3). The following covariates were adjusted for in Model 2: parents' age (30–39, 40–49, 50 years or older) at the time of bereavement, educational level (basic, upper, higher) as by December 31 the year before the child's cancer diagnosis, marital status (married; not married) as by December 31 the year before the child's cancer diagnosis, country of birth (Sweden; Other), and time since diagnosis to death. History of mental health problems was added as a covariate in Model 3. To assess the robustness of the results, sensitivity analyses were performed by stratifying analyses by history of mental health problems (yes/no) and examining the ORs of the total sample to that of the restricted sample of bereaved parents without a history of mental health problems. Specifically, we wanted to examine if bereavement was associated with significantly higher odds of prescriptions of psychotropic medication also among bereaved mothers/fathers without a history of mental health problems or if higher odds observed from the main models were mainly attributed a history of mental health problems. All analyses were carried out in relation to time of bereavement, where year 0 (index) is the year of bereavement (calculated as the date of the child's death +365 days).

## 3 RESULTS

### 3.1 Study sample

Data from 184 mothers and 184 fathers of 184 children diagnosed with cancer in adolescence and who had died between 1 July 2006 and 31 December 2014 were included. Non‐bereaved referents were 1652 mothers and 1639 fathers of cancer‐free adolescent comparisons. For the majority (90%) of bereaved parents, eight or more non‐bereaved parents of cancer‐free controls were included. The least number of identified non‐bereaved parents was six (the case for three bereaved mothers; four bereaved fathers).

Demographic information for the sample can be found in Table 1. Median age of the adolescents at death was 19 years (range: 14–32) and median time between cancer diagnosis and death was 2 years (range: 0–17). Most parents (72%) lost their child during the first 3 years after diagnosis and a small proportion (7%) lost their child 10+ years after diagnosis. The largest proportion of mothers (50%) were 40–49 years of age at the time of bereavement whereas the majority (59%) of fathers were 50 years or older. Moreover, the majority had at least an upper secondary education, were married, born in Sweden and had no history of mental health problems. Compared to non‐bereaved mothers, more bereaved mothers had a history of mental health problems before the child's cancer diagnosis (Table 1).

**TABLE 1:** Characteristics of the bereaved parents of adolescents with cancer and of non‐bereaved parents of cancer‐free comparisons

	Bereaved parents		Non‐bereaved parents		*p*‐value (χ^2^) for difference between bereaved and non‐bereaved
	Mothers (*n* = 184)	Fathers (*n* = 184)	Mothers (*n* = 1652)	Fathers (*n* = 1639)	Mothers/fathers
Age at bereavement/index, *n* (%)					
30–39	14 (8)	3 (2)	118 (7)	39 (2)	0.255/0.344
40–49	91 (50)	72 (39)	921 (56)	718 (44)	
50+	79 (43)	109 (59)	613 (37)	882 (54)	
Education, *n* (%)					
Basic (0–9 years)	19 (10)	27 (15)	176 (11)	296 (18)	0.343/0.241
Upper (10–12 years)	98 (53)	103 (56)	848 (51)	821 (50)	
Higher (13+ years)	62 (34)	48 (26)	608 (37)	489 (30)	
Marital status, *n* (%)					
Married^a^	97 (53)	100 (54)	946 (57)	962 (59)	0.088/0.343
Not married^b^	82 (45)	78 (42)	689 (42)	644 (39)	
Country of birth, *n* (%)					
Sweden	161 (88)	155 (84)	1429 (86)	1392 (85)	0.792/0.889
Other	23 (12)	29 (16)	223 (14)	247 (15)	
History of mental health problems^c^, *n* (%)					
No	161 (88)	170 (92)	1529 (93)	1529 (93)	0.024/0.761
Yes	23 (12)	14 (8)	123 (7)	110 (7)	

*Note*: Percentages might not add up to 100 due to rounding and missing information.

^a^ Includes registered partner (one mother in the general population sample).

^b^ Includes widow/widower (three bereaved mothers, two bereaved fathers, seven non‐bereaved mothers and four non‐bereaved fathers).

^c^ Defined as any in‐patient or out‐patient psychiatric diagnosis during the 5 years prior to date of the child's cancer diagnosis/index for parents in the general population sample.

### 3.2 Psychotropic medication

**FIGURE 1:**
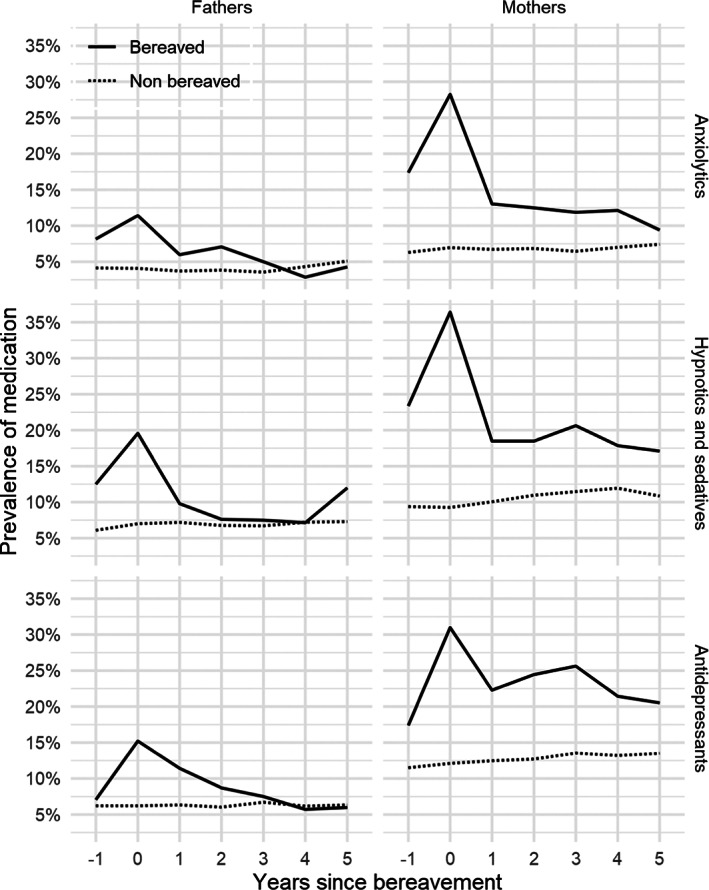
Proportion of bereaved and non‐bereaved father and mothers, respectively, with a prescription of a psychotropic medication (N05B, N05C and N06A) during the study period. Year 0 = the year of the child's death.

The highest proportion of mothers and fathers with prescriptions of either class of psychotropic medication was found for the year of the child's death, followed by the year preceding and the year after the child's death (Figure 1). At the year of the child's death, about 28%–36% (*n* = 52–67) of mothers and 11%–20% of fathers (*n* = 21–36) had a prescription of anxiolytics, hypnotics/sedatives or antidepressants. The corresponding percent for non‐bereaved mothers and fathers was around 7%–12% (*n* = 115–200) and 4%–7% (*n* = 67–115), respectively (Figure 1). As illustrated in Figure 1, for each class of medication, there was a marked decrease in the proportion of bereaved mothers with a prescription from the year of the child's death to relatively stable prevalence rates 1–5 years after the child's death (9%–26%, *n* = 11–45, depending on class of medication), yet remaining higher than non‐bereaved mothers over the study period. Likewise, the proportion of bereaved fathers with a prescription decreased markedly from the year after the child's death, with relatively stable prevalence rates, similar to that of non‐bereaved fathers, around 5%–10% (*n* = 4–21, depending on class of medication) 1–5 years after the child's death.

The cumulative incidence of a prescription of anxiolytics, hypnotics/sedatives or antidepressants during the study period (1 year prior to 5 years after bereavement) for cancer‐bereaved mothers was 62% (*n* = 114; 95% CI, 54–69), compared with 37% (*n* = 611; 95% CI, 35–39) in non‐bereaved mothers. The corresponding percent for bereaved fathers was 38% (*n* = 69; 95% CI, 31–45), compared with 25% (*n* = 417; 95% CI, 23–28) in non‐bereaved fathers. Table 2 presents the cumulative incidence for each class of medication.

**TABLE 2:** The cumulative incidence of first prescription of anxiolytics, hypnotics/sedatives or antidepressants for the study period (1 year prior up to 5 years after the child's death) in cancer‐bereaved and non‐bereaved mother and fathers, respectively

	Anxiolytics (N05B)				Hypnotics/sedatives (N05C)				Antidepressants (N06A)			
	Bereaved		Non‐bereaved		Bereaved		Non‐bereaved		Bereaved		Non‐bereaved	
	*n*	% (95% CI)	*n*	% (95% CI)	*n*	% (95% CI)	*n*	% (95% CI)	*n*	% (95% CI)	*n*	% (95% CI)
Mothers	72	39 (32–47)	314	19 (17–21)	87	47 (40–55)	389	24 (22–26)	77	42 (35–49)	366	22 (20–24)
Fathers	36	20 (14–26)	198	12 (11–14)	51	28 (22–35)	267	16 (15–18)	38	21 (15–27)	205	13 (11–14)

Table 3 shows the results of the crude and adjusted regression models for each class of medication in mothers. Among mothers, when taking into account sociodemographic factors and history of mental health problems, bereavement was associated with statistically significant higher odds of prescriptions of anxiolytics and hypnotics/sedatives from the year preceding the child's death: (OR_adj_: 2.78, 95% CI, 1.84–4.21 and OR_adj_: 2.75, 95% CI, 1.93–3.93, respectively) to four (anxiolytics: OR_adj_: 1.81, 95% CI, 1.04–3.14) and five (hypnotics/sedatives: OR_adj_ 1.92, 95% CI, 1.24–2.99) years after bereavement (Table 3). Statistically significant higher odds of prescriptions of antidepressants were also seen from the year of the child's death to 5 years after bereavement (Model 3, Year 0: OR_adj_ 3.07, 95% CI, 2.11–4.45 to Year 5: OR_adj_: 1.67, 95% CI, 1.08–2.57). In relation to time since the child's death, for all three classes of medication, the highest OR was detected at the year of the child's death (Model 3: anxiolytics; OR_adj_: 5.67, 95% CI, 3.78–8.52; hypnotics/sedatives; OR_adj_: 5.49, 95% CI, 3.78–7.97; antidepressants: OR_adj_: 3.07, 95% CI, 2.11–4.45). Mothers who were unmarried, who were born outside Sweden or who had a history of mental health problems were more likely to have a prescription of psychotropic medication, whereas mothers with a higher education were found less likely to be prescribed psychotropic medication (Table 3).

**TABLE 3:** Prescription of psychotropic medication (crude and adjusted odds ratio [OR] with 95% confidence internals [CIs]) in bereaved mothers 1 year before and 5 years after the child's death, by medication type (anxiolytics, hypnotics/sedatives, and antidepressants)

	Anxiolytics (N05B)			Hypnotics/sedatives (N05C)			Antidepressants (N06A)		
	Model 1 (crude)	Model 2	Model 3	Model 1 (crude)	Model 2	Model 3	Model 1 (crude)	Model 2	Model 3
	OR (95% CI)	OR (95% CI)	OR (95% CI)	OR (95% CI)	OR (95% CI)	OR (95% CI)	OR (95% CI)	OR (95% CI)	OR (95% CI)
Time since child's death^a^									
Year −1	**2.81 (1.94–4.09)**	**2.85 (1.93–4.22)**	**2.78 (1.84–4.21)**	**2.99 (2.17–4.10)**	**3.02 (2.18–4.19)**	**2.75 (1.93–3.93)**	**1.53 (1.09–2.15)**	**1.49 (1.05–2.13)**	1.32 (0.90–1.95)
Year 0 (year of child's death)	**5.27 (3.63–7.64)**	**5.48 (3.73–8.05)**	**5.67 (3.78–8.52)**	**5.61 (3.98–7.91)**	**5.73 (4.04–8.13)**	**5.49 (3.78–7.97)**	**3.26 (2.31–4.60)**	**3.26 (2.29–4.63)**	**3.07 (2.11–4.45)**
Year 1	**2.00 (1.30–3.09)**	**2.01 (1.28–3.16)**	**1.91 (1.19–3.08)**	**2.22 (1.60–3.08)**	**2.24 (1.60–3.13)**	**1.98 (1.38–2.85)**	**2.08 (1.58–2.74)**	**2.06 (1.55–2.73)**	**1.87 (1.37–2.55)**
Year 2	**1.91 (1.23–2.97)**	**1.92 (1.21–3.04)**	**1.79 (1.09–2.92)**	**2.22 (1.60–3.08)**	**2.24 (1.60–3.14)**	**2.01 (1.40–2.87)**	**2.35 (1.74–3.17)**	**2.34 (1.71–3.20)**	**2.15 (1.53–3.03)**
Year 3	**1.73 (1.07–2.81)**	**1.73 (1.05–2.85)**	1.59 (0.93–2.71)	**2.51 (1.75–3.60)**	**2.54 (1.76–3.68)**	**2.33 (1.56–3.47)**	**2.24 (1.59–3.17)**	**2.23 (1.56–3.20)**	**2.06 (1.40–3.04)**
Year 4	**1.94 (1.19–3.16)**	**1.93 (1.16–3.21)**	**1.81 (1.04–3.14)**	**2.10 (1.40–3.13)**	**2.12 (1.41–3.18)**	**1.92 (1.24–2.99)**	**1.92 (1.32–2.79)**	**1.91 (1.29–2.81)**	**1.75 (1.15–2.65)**
Year 5	1.55 (0.89–2.70)	1.51 (0.85–2.71)	1.40 (0.75–2.62)	**2.08 (1.35–3.19)**	**2.09 (1.35–3.22)**	**1.92 (1.20–3.07)**	**1.83 (1.24–2.69)**	**1.81 (1.22–2.70)**	**1.67 (1.08–2.57)**
Age (years) of parent									
40–49 vs 30–39 (ref)		0.71 (0.46–1.09)	0.84 (0.53–1.31)		0.74 (0.49–1.11)	0.88 (0.59–1.31)		0.71 (0.48–1.07)	0.84 (0.56–1.26)
50+ vs 30–39 (ref)		0.67 (0.41–1.08)	0.81 (0.49–1.33)		0.95 (0.61–1.49)	1.14 (0.74–1.76)		0.76 (0.49–1.19)	0.90 (0.57–1.40)
Marital status									
Not married vs married (ref)		**1.52 (1.19–1.95)**	**1.39 (1.08–1.78)**		1.17 (0.92–1.48)	1.09 (0.86–1.38)		**1.43 (1.13–1.80)**	**1.34 (1.06–1.71)**
Education									
Upper vs basic (ref)		**0.60 (0.41–0.86)**	0.71 (0.49–1.03)		**0.63 (0.44–0.90)**	0.72 (0.50–1.03)		0.72 (0.50–1.02)	0.83 (0.57–1.21)
Higher vs basic (ref)		**0.44 (0.29–0.66)**	**0.52 (0.35–0.78)**		**0.63 (0.43–0.92)**	0.74 (0.50–1.07)		**0.61 (0.41–0.89)**	0.72 (0.48–1.07)
Country of birth									
Other vs Sweden (ref)		**1.65 (1.19–2.28)**	**1.51 (1.08–2.12)**		1.18 (0.86–1.62)	1.08 (0.78–1.48)		1.05 (0.76–1.44)	0.97 (0.70–1.35)
History of mental health problems^b^									
Yes vs no (ref)			**6.01 (4.38–8.24)**			**4.96 (3.64–6.76)**			**5.13 (3.72–7.07)**
Time since diagnosis to death, years		0.99 (0.95–1.03)	0.99 (0.95–1.03)		1.00 (0.96–1.03)	1.00 (0.96–1.03)		0.99 (0.96–1.03)	0.99 (0.96–1.03)

*Note*: Bold values denote statistical significance at the *p* < 0.05 level.

Abbreviations: OR, odds ratio; Ref, reference category.

^a^ Reference time is non‐bereaved mothers year 0 (date of child's death plus 365 days).

^b^ Defined as any in‐patient or out‐patient psychiatric diagnosis during the 5 years prior to date of the child's cancer diagnosis/inclusion for non‐bereaved parents in the general population sample.

Table 4 shows the results of the crude and adjusted regression models for each class of medication in fathers. Among fathers, when taking into account sociodemographics and history of mental health problems, bereavement was associated with a statistically significant higher prevalence of use of anxiolytics and hypnotics/sedatives at the year before the child's death (OR_adj_: 2.05, 95% CI, 1.16–3.62 and OR_adj_: 1.93, 95% CI, 1.32–2.82, respectively) and at the year of bereavement (OR_adj_: 3.08, 95% CI, 1.81–5.23 and OR_adj_: 3.39, 95% CI, 2.22–5.17, respectively), and for antidepressants from the year of up to the year after the child's death, (Year 0: OR_adj_: 2.87, 95% CI, 1.76–4.68; Year 1: OR_adj_: 1.98, 95% CI, 1.33–2.96) (Table 4). In relation to time since the child's death, for all three classes of medication, bereaved fathers showed the highest OR at the year of the child's death (Model 3: anxiolytics; OR_adj_: 3.08, 95% CI, 1.81–5.23; hypnotics/sedatives; OR_adj_: 3.39, 95% CI, 2.22–5.17; antidepressants: OR_adj_: 2.87, 95% CI, 1.76–4.68). At 2 years after the child's death, the odds of having a prescription of any of the studied psychotropic medications were the same for bereaved and non‐bereaved fathers. Unmarried fathers and fathers with a history of mental health problems were more likely to have a prescription of psychotropic medication (Table 4).

**TABLE 4:** Prescription of psychotropic medication (crude and adjusted odds ratio [OR] with 95% confidence internals [CIs]) in bereaved fathers 1 year before and five after the child's death, by medication type (anxiolytics, hypnotics/sedatives, and antidepressants)

	Anxiolytics (N05B)			Hypnotics/sedatives (N05C)			Antidepressants (N06A)		
	Model 1 (crude)	Model 2	Model 3	Model 1 (crude)	Model 2	Model 3	Model 1 (crude)	Model 2	Model 3
	OR (95% CI)	OR (95% CI)	OR (95% CI)	OR (95% CI)	OR (95% CI)	OR (95% CI)	OR (95% CI)	OR (95% CI)	OR (95% CI)
Time since child's death^a^									
Year −1	**2.08 (1.24–3.49)**	**2.07 (1.23–3.49)**	**2.05 (1.16–3.62)**	**1.89 (1.32–2.72)**	**1.91 (1.32–2.75)**	**1.93 (1.32–2.82)**	1.15 (0.75–1.76)	1.10 (0.70–1.72)	1.12 (0.71–1.78)
Year 0 (year of child's death)	**3.02 (1.81–5.07)**	**3.04 (1.80–5.10)**	**3.08 (1.81–5.23)**	**3.22 (2.14–4.86)**	**3.27 (2.15–4.96)**	**3.39 (2.22–5.17)**	**2.71 (1.73–4.24)**	**2.64 (1.68–4.16)**	**2.87 (1.76–4.68)**
Year 1	1.49 (0.80–2.78)	1.48 (0.79–2.77)	1.41 (0.71–2.81)	1.44 (0.92–2.24)	1.45 (0.93–2.26)	1.46 (0.92–2.31)	**1.94 (1.36–2.78)**	**1.88 (1.30–2.72)**	**1.98 (1.33–2.96)**
Year 2	1.78 (1.00–3.19)	1.77 (0.99–3.18)	1.72 (0.91–3.26)	1.09 (0.63–1.89)	1.10 (0.63–1.91)	1.10 (0.62–1.92)	1.44 (0.86–2.38)	1.39 (0.82–2.35)	1.42 (0.82–2.48)
Year 3	1.17 (0.56–2.45)	1.16 (0.56–2.40)	1.11 (0.50–2.48)	1.01 (0.56–1.82)	1.02 (0.57–1.83)	1.02 (0.56–1.87)	1.30 (0.77–2.21)	1.26 (0.73–2.17)	1.29 (0.71–2.33)
Year 4	0.86 (0.38–1.94)	0.85 (0.38–1.90)	0.81 (0.34–1.90)	1.02 (0.55–1.89)	1.02 (0.55–1.89)	1.03 (0.55–1.91)	1.22 (0.74–2.03)	1.18 (0.70–1.99)	1.20 (0.69–2.08)
Year 5	1.07 (0.48–2.38)	1.06 (0.47–2.36)	0.99 (0.42–2.31)	1.58 (0.92–2.70)	1.60 (0.93–2.75)	1.61 (0.93–2.80)	1.17 (0.61–2.26)	1.14 (0.58–2.24)	1.12 (0.56–2.25)
Age (years) of parent									
40–49 vs. 30–39 (ref)		0.72 (0.32–1.60)	0.73 (0.32–1.65)		0.75 (0.36–1.58)	0.76 (0.37–1.56)		0.91 (0.33–2.49)	0.94 (0.34–2.65)
50+ vs. 30–39 (ref)		0.68 (0.29–1.55)	0.64 (0.28–1.47)		0.80 (0.38–1.69)	0.77 (0.37–1.59)		0.90 (0.32–2.52)	0.86 (0.30–2.46)
Marital status									
Not married vs. married (ref)		**1.41 (1.02–1.96)**	1.09 (0.78–1.53)		**1.57 (1.20–2.06)**	**1.34 (1.01–1.78)**		**1.39 (1.02–1.89)**	1.07 (0.77–1.48)
Education									
Upper vs. basic (ref)		1.06 (0.69–1.63)	1.08 (0.70–1.69)		1.03 (0.71–1.51)	1.06 (0.72–1.54)		1.23 (0.76–2.01)	1.30 (0.79–2.13)
Higher vs. basic (ref)		0.63 (0.38–1.05)	0.75 (0.45–1.26)		1.02 (0.68–1.55)	1.15 (0.76–1.75)		0.75 (0.44–1.28)	0.87 (0.51–1.51)
Country of birth									
Other vs. Sweden (ref)		1.41 (0.94–2.12)	1.13 (0.74–1.71)		0.90 (0.62–1.31)	0.76 (0.53–1.10)		**1.58 (1.08–2.33)**	1.23 (0.82–1.84)
History of mental health problems^b^									
Yes vs. no (ref)			**6.91 (4.69–10.19)**			**4.61 (3.19–6.66)**			**7.95 (5.46–11.58)**
Time since diagnosis to death, years		1.00 (0.95–1.05)	1.01 (0.96–1.07)		1.00 (0.96–1.04)	1.01 (0.97–1.05)		0.97 (0.93–1.02)	0.98 (0.94–1.03)

*Note*: Bold values denote statistical significance at the *p* < 0.05 level.

Abbreviations: OR, odds ratio; Ref, Reference category.

^a^ Reference time is non‐bereaved fathers year 0 (date of child's death plus 365 days).

^b^ Defined as any in‐patient or out‐patient psychiatric diagnosis during the 5 years prior to date of the child's cancer diagnosis/inclusion for non‐bereaved parents in the general population sample.

Sensitivity analyses restricted to bereaved parents without history of mental health problems (161 mothers; 170 fathers) showed similar results as the main models shown in Tables 3 and 4, except that bereaved mothers without a history of mental health problems showed no statistically significant higher odds than non‐bereaved mothers of having a prescription hypnotics/sedatives at 5 years after the child's death (OR_adj_: 1.77, 95% CI: 1.00–3.15), and bereaved fathers without a history of mental health problems showed statistically significant higher odds than non‐bereaved fathers of prescriptions of antidepressants at 3 years after the child's death (OR_adj_: 1.87, 95% CI: 1.05–3.32).

## 4 DISCUSSION

This is the first register‐based study of psychotropic medication prescriptions in bereaved parents of children diagnosed with cancer during adolescence. The results showed a higher use of prescribed psychotropic medication in bereaved mothers and fathers compared to non‐bereaved parents. The highest odds were found the year before and of bereavement, with a higher prevalence of use over several years in mothers. At 5 years after the child's death, bereaved mothers showed higher odds of having a prescription of hypnotics/sedatives and antidepressants, while bereaved fathers showed similar odds as non‐bereaved fathers for the studied psychotropic medications at two years after the child's death.

Psychotropic medication may be used as a mean to alleviate responses to bereavement. The results of this study show that around the time of bereavement, about one third of mothers and one fifth of fathers have a prescription of anxiolytics, hypnotics/sedatives or antidepressants. These estimates are high compared to the non‐bereaved parents, likely reflecting the fact that a substantial subgroup of bereaved parents experience emotional reactions intense enough to warrant medical treatment. Both mothers and fathers showed a higher prevalence of prescriptions already before the loss, indicating distress associated with seeing their child suffer from the disease and treatment, but also that the grief process may have started well before the child's actual death. This can be understood as anticipatory grief and that parents are in need of psychotropic medication to cope with the child's impending death. In the context of childhood cancer, the outcome is often uncertain and many parents recognize the possibility of their child's death at the time of diagnosis. We have previously shown that parents of survivors of adolescent cancer (excluding bereaved parents) are at increased risk of use of anxiolytics and sedatives in the immediate post‐diagnostic phase.^24^ The results of the present study might thus reflect parents' reactions to the initial cancer diagnosis as well. Aligned with this, anticipatory grief responses have been reported by parents of children newly diagnosed with cancer.^41^

Our results indicate that cancer‐bereaved parents are at risk for clinical levels of psychological distress, but also demonstrate a move toward ‘baseline’ as regards prescription of psychotropic medication over time, which may illustrate the process of adaptation to the child's death. Consistent with previous research on psychotropic medication use in parents following child loss from disease or external cause,^12^ we found that bereaved mothers and fathers show different patterns, with mothers being at increased risk also in the long term. This might reflect an increased vulnerability in mothers due to biological factors and/or gender‐related norms and role expectations, seen for example in mothers of children with cancer who often take on the primary caregiver role.^42^ The higher prevalence of use compared to non‐bereaved parents was evident up to 4 and 5 years after the loss among mothers depending on class of medication, and up to 1 year after the loss among fathers. The longest period of higher odds, evident for the entire study period of 6 years, was found for hypnotics/sedatives, indicating a high prevalence of clinical levels of sleep disturbances over several years. This is consistent with a study where parents reported elevated symptoms of insomnia 1–5 years after the loss of a child to cancer.^7^

To recognize and support parents is an established standard of pediatric oncology psychosocial care, and this includes palliative and bereavement support.^43^, ^44^ The results of this study, showing that cancer‐bereaved parents, in particular mothers, are in need of psychotropic medication for a long period of time, underscores the importance of implementing these standards and to consider also non‐pharmacological forms of treatments, for example psychotherapy.^45^, ^46^, ^47^ As highlighted by our results, available bereavement support should include follow‐up contact and support from the healthcare team and screening of parents at risk for adverse bereavement outcomes.^44^, ^48^ In contrast to findings of a recent questionnaire‐based study,^15^ a history of mental health problems was associated with increased likelihood of psychotropic medication use after bereavement. This is however also supported by other studies showing that past psychological distress is a risk factor during grief.^49^, ^50^ Then again, the increased risk of use among parents with a history of mental health problems might also be related to these parents already being connected with the healthcare system, thus having easier access to treatment. Nevertheless, a history of mental health problems should be acknowledged as a predisposing factor to vulnerability. Moreover, we found that mothers with a low education, born outside Sweden and mothers and fathers who were not married were at particular risk. Both underutilization and higher use of psychiatric care, depending on time spent in Sweden, have been observed in migrants.^51^ Our data do not allow for such conclusions to be drawn, but the observed higher odds among foreign‐born mothers may suggest more pronounced mental health problems in response to bereavement among bereaved mothers born outside Sweden compared to bereaved mothers born in Sweden. This could be related to lower access to psychosocial support (e.g., counseling or psychotherapy) provided by the healthcare, cultural differences and/or language barriers,^52^, ^53^ but the finding warrants further investigation. Our results furthermore highlight non‐married parents as a vulnerable group. This finding is in line with a previous report on mothers of children with cancer in treatment,^54^ but has to our knowledge not been acknowledged as a risk factor for use of psychotropic medication in parents of children with cancer.^21^

Strengths of this study include the use of high‐quality registers, a population‐based sample with a matched general population sample, and possibility to take parents' socioeconomic position and history of mental health problems into account for more accurate estimates. An important limitation is that we do not have information on the specific indication of the prescribed medication and thus we cannot create a direct link between the medication and the symptoms that the medication was intended for. Still, the primary indications of the studied medications are well known and correspond to psychological distress reported by parents themselves.^17^, ^18^, ^19^, ^20^ Moreover, prescription of psychotropic medication should be conditioned by needs. The concept of ‘need’ is however ambiguous, and care‐seeking and access are influenced by factors other than mental health disorders. Accordingly, while register data on psychotropic medication has shown to be a suitable proxy for mental health,^12^ increased use of psychotropic medications may not be directly equivalent to increased psychological distress. The normalization of symptoms of anxiety, depression, and sleep disturbances among fathers as indicated by our results is somewhat shorter than found in questionnaire‐based studies showing more psychological distress in bereaved parents up to 8–9 years after the loss.^7^, ^15^, ^18^ Aligned with the above line of reasoning, no use of psychotropic medication does not necessarily equal no distress. Subclinical levels of psychological distress are better captured by self‐reports than register data on prescriptions of medications. Furthermore, our results show a decrease in prevalence of use also among mothers, but we did not see a return to levels comparable to non‐bereaved mothers in the general population during follow‐up. Further studies with longer follow‐up are needed to determine the pattern of use of psychotropic medication in mothers in the longer term, when more than 5 years have passed since bereavement. Moreover, data that allow distinguishing partners within parental dyads would also allow future work to expand on the results of this study to account for possible interdependence in psychotropic medication use between parental dyads.

## 5 CONCLUSIONS

The results of this study provide insights into the pattern of use of psychotropic medication among parents who lose a child to cancer. Indicative of mental health problems of clinical importance, cancer‐bereaved parents had a higher prevalence of use of psychotropic medication before and after bereavement. A decrease in medication use was evident with time, but mothers displayed a higher use compared to non‐bereaved mothers still at 5 years after bereavement, while fathers showed no difference to non‐bereaved fathers after 2 years. Our results point to a need for continued follow‐up to identify and support parents at risk of adverse long‐term bereavement outcomes.

## AUTHOR CONTRIBUTIONS

**Emma Hovén:** Conceptualization (equal); formal analysis (equal); investigation (equal); methodology (equal); validation (equal); visualization (equal); writing – original draft (lead); writing – review and editing (equal). **Lisa Ljungman:** Conceptualization (equal); funding acquisition (equal); methodology (equal); writing – original draft (equal); writing – review and editing (equal). **Josefin Sveen:** Methodology (equal); writing – review and editing (equal). **Charlotte Skoglund:** Methodology (equal); writing – review and editing (equal). **Gustaf Ljungman:** Methodology (equal); writing – review and editing (equal). **Rickard LJUNG:** Conceptualization (equal); methodology (equal); writing – review and editing (equal). **Anna Wikman:** Conceptualization (equal); formal analysis (equal); funding acquisition (lead); investigation (equal); methodology (equal); project administration (lead); supervision (lead); validation (equal); visualization (equal); writing – original draft (equal); writing – review and editing (equal).

## FUNDING INFORMATION

The Swedish Childhood Cancer Fund (PR2019‐0094; TJ2019‐0045).

## CONFLICT OF INTEREST

Dr. Rickard Ljung is employed at the Swedish Medical Products Agency, SE‐751 03 Uppsala, Sweden. The views expressed in this paper do not necessarily represent the views of the Government agency.

## ETHICS STATEMENT

This study was approved by the Regional Ethical Review Board, Uppsala, Sweden (approval number 2017/117). Data confidentiality was approved by the National Board of Health and Welfare and Statistics Sweden.

## Data Availability

The data are not publicly available due to ethical restrictions. The data that support the findings of this study are available from the corresponding author, upon reasonable request and with necessary ethics approval
